# Gaming through the pain: psychological flow and self-efficacy amid musculoskeletal challenges in eSports

**DOI:** 10.3389/fpsyg.2025.1640559

**Published:** 2025-09-12

**Authors:** Samiah Alqabbani, Reema Alhussaini, Shumukh Alsaedan, Manar Alfaqi, Reema Almudaifer, Najd Zainaldeen, Hanan Alsaeed, Maha Algabbani, Wafa Alahmari, Afrah Almuwais, Madawi Alotaibi

**Affiliations:** ^1^Department of Rehabilitation Sciences, College of Health and Rehabilitation Sciences, Princess Nourah Bint Abdulrahman University, Riyadh, Saudi Arabia; ^2^Department of Rehabilitation Health Sciences, College of Applied Medical Sciences, King Saud University, Riyadh, Saudi Arabia

**Keywords:** eSports, musculoskeletal pain, flow state, self-efficacy, gaming performance, digital athletes, Saudi Arabia

## Abstract

**Introduction:**

The rapid expansion of eSports has highlighted concerns regarding the physical and psychological well-being of professional gamers, which have intensified.

**Methods:**

This study examined the prevalence of Musculoskeletal (MSK) pain and its association with flow states and self-efficacy among two hundred and thirty-two male professional gamers registered with the Saudi eSports Federation. Participants completed validated tools: the Extended Nordic Musculoskeletal Questionnaire, Flow 4D16, and General Self-Efficacy Scale (GSE-3). Descriptive and multivariate analyses were used.

**Results:**

MSK pain was highly prevalent (72%), especially in the neck (38.4%), lower back (31.5%), and wrists/hands (24.1%). Logistic regression identified input tool and device category as significant predictors of shoulder pain (*p* < 0.05). Despite widespread pain, flow and self-efficacy scores remained moderate to high, with no significant associations between psychological outcomes and MSK pain (MANCOVA, Pillai's Trace = 0.044, *p* = 0.069).

**Discussion:**

These findings suggest a degree of psychological resilience among eSports athletes, underscoring the complexity of physical-psychological interplay in gaming contexts. Future studies should explore mediating factors such as pain coping, motivation, and recovery behaviors.

## Introduction

The eSports industry has witnessed unprecedented global growth in recent years, and Saudi Arabia has rapidly emerged as a regional leader in this digital revolution. The Kingdom's commitment to fostering its eSports sector is underscored by Vision 2030, the launch of the National Gaming and ESports Strategy (NGES), and the establishment of the Saudi eSports Federation (SEF), which leads the development, governance, and promotion of eSports across the country (Saudi eSports Federation, 2023). These efforts aim to position Saudi Arabia as a global hub for competitive gaming, projected to contribute $13.3 billion to the GDP and create 39,000 new jobs by 2030 (Pw and Middle East, 2023). Additionally, the ESports World Cup, set to be held annually in Riyadh starting in 2024, further reflects the nation's ambition to become a world leader in professional gaming (ESports World Cup Foundation, 2024).

While these developments mark major economic and cultural achievements, they also raise pressing questions about the cognitive and physical demands placed on eSports athletes. Extended gameplay, often involving prolonged sitting, repetitive hand movements, and improper ergonomics, exposes players to a high risk of Musculoskeletal (MSK) disorders (Tholl et al., 2022). In Saudi Arabia, 86.2% of competitive gamers reported MSK symptoms, with pain most commonly affecting the hands, neck, shoulders, and lower back (Fathuldeen et al., 2023). These local findings mirror global trends, as research consistently highlights the impact of gaming on physical health. Studies have identified similar patterns in the prevalence of musculoskeletal pain among gamers, particularly in the neck and lower back (Yona et al., 2020; Forman and Holmes, 2023).

The implications of MSK pain extend beyond physical discomfort into the domain of performance psychology. Pain can negatively influence sensorimotor coordination, reduce attentional bandwidth, and interfere with working memory—all essential faculties for high-level eSports performance (Kim et al., 2021; Phelps, 2006). It may also disrupt training, increase reliance on pain medications, or even force withdrawal from competitions (Lindberg et al., 2020; Emara et al., 2020). Chronic MSK symptoms are further associated with fatigue, sleep disruption, and decreased quality of life, particularly among digital professionals and gamers (Demissie et al., 2024).

Cognitive performance in eSports is also influenced by psychological variables such as flow and self-efficacy. Flow state—defined as a deep state of task immersion and automaticity—is widely recognized as a determinant of optimal performance under pressure (Khoshnoud et al., 2020; Pavlas et al., 2010a,b). Clear goals, immediate feedback, and the balance between challenge and skill facilitate it. However, pain and fatigue may act as disruptive stimuli that hinder the maintenance of flow states (Ditchburn et al., 2020; Martiny et al., 2023). Likewise, self-efficacy, or one's belief in their capacity to succeed, underpins persistence, coping, and adaptive effort regulation (Gutierrez, 2021). Gamers with high self-efficacy are better equipped to handle performance challenges (Luszczynska et al., 2005), but ongoing physical discomfort may progressively erode these beliefs, compromising sustained performance (Lindberg et al., 2020; Ditchburn et al., 2020).

To interpret the interplay between MSK pain and psychological functioning, this study draws upon three complementary theoretical frameworks: the biopsychosocial model, Attentional Control Theory, and Bandura's Self-Efficacy Theory. The biopsychosocial model, as outlined by (Gatchel et al. 2007), conceptualizes pain not merely as a physiological event but as a multidimensional construct shaped by psychological and social influences. This perspective emphasizes that cognitive and emotional factors may buffer the impact of physical discomfort on performance. Complementing this, the Attentional Control Theory (Eysenck et al., 2007) proposes that pain can disrupt goal-directed attention by increasing cognitive load, potentially interfering with immersion in task-related activities such as gameplay. Additionally, Bandura's Self-Efficacy Theory (1997) highlights the role of belief in one's ability to manage challenges—including physical strain—as a critical determinant of motivation, resilience, and sustained performance. Together, these frameworks provide a nuanced understanding of how Musculoskeletal pain may impact cognitive immersion (flow) and perceived competence (self-efficacy) in high-performance eSports contexts.

Despite growing international research on these topics, limited attention has been given to the intersection of MSK disorders, flow, and self-efficacy—particularly among professional gamers in Saudi Arabia. Given the uniquely immersive, cognitively demanding nature of competitive eSports, and the Kingdom's leadership role through initiatives like SEF and the eSports World Cup, addressing this gap is essential to inform evidence-based health and performance strategies.

Based on prior research suggesting that physical discomfort can disrupt attentional processes and self-regulation, it was hypothesized that the presence of musculoskeletal pain would be associated with lower scores in both flow and self-efficacy. Specifically, professional gamers experiencing pain—particularly in the neck, back, or upper limbs—were expected to report significantly reduced cognitive flow and self-efficacy compared to those without pain (H1). The null hypothesis (H0) assumed no significant differences between groups. Therefore, this study aims to investigate the prevalence and consequences of musculoskeletal pain among professional gamers in Saudi Arabia and explore its potential association with flow states and self-efficacy during gameplay.

## Methods

### Study design and setting

This study adopted a cross-sectional, descriptive design and was conducted in the Kingdom of Saudi Arabia. Data were collected from March to April 2024 using a self-administered online questionnaire hosted on Microsoft Forms.

### Target population and sampling procedure

The target population consisted of professional gamers in Saudi Arabia. Participants were included if they were aged 18 years or older, officially registered with SEF, and actively involved in competitive eSports for monetary prizes or awards. At the time of data collection, the official registry consisted exclusively of male professional players, and thus no female gamers were available for inclusion. Individuals were excluded if they had a history of fractures within the past six months, or if they had any pre-existing medical conditions, recent surgeries, physical disabilities, or cognitive impairments that could interfere with survey participation or bias musculoskeletal symptom reporting.

### Data collection instrument

Data were collected using a structured, self-administered online questionnaire composed of four sections. Prior to data collection, the digital version of the questionnaire was reviewed by five individuals to assess the clarity, logic, and branching functionality of the items. Based on their feedback, modifications were made to improve wording and navigation. Once the issues were resolved, the revised form was distributed to a small group of amateur gamers to ensure usability and completeness before disseminating to the target professional population.

### Demographic information

The first section captured basic demographic and gaming-related variables, including age, gender, type of gaming device used, average daily gaming hours, and years of competitive gaming experience.

### Extended Nordic Musculoskeletal Questionnaire (NMQ-E)

The Extended Nordic Musculoskeletal Questionnaire (NMQ-E) is a validated instrument designed to assess musculoskeletal symptoms across nine anatomical regions: the neck, upper back, lower back, shoulders, elbows, wrists/hands, hips, knees, and ankles/feet (Dawson et al., 2009). It evaluates symptom prevalence at four time points—lifetime, annual, monthly, and point prevalence—using a binary response format (“Yes” or “No”). A body map diagram is included to help participants accurately identify areas of discomfort. The questionnaire also assesses the functional consequences of MSK pain, including its impact on daily or competitive activity, healthcare visits, medication usage, absenteeism, and hospitalizations. The Arabic version of the NMQ-E has been previously validated and demonstrated strong content and construct validity, as well as high test-retest reliability (Al Amer and Alharbi, 2023). This tool is widely used in research involving occupational and sport-related musculoskeletal disorders.

### Flow State Scale (FSS)

The Flow State Scale (FSS) is used to measure the degree to which individuals experience flow—a psychological state characterized by deep immersion, intrinsic motivation, and optimal engagement. This study used the Arabic version of the FSS based on the Flow 4D16 model, which includes sixteen items across four dimensions: cognitive flow, time distortion, ego detachment, and wellbeing (Chalghaf et al., 2019). Participants responded to each item using a 5-point Likert scale ranging from 1 (“strongly disagree”) to 5 (“strongly agree”). Higher scores reflect stronger flow experiences. The FSS has demonstrated solid internal consistency and construct validity in Arabic-speaking contexts and is particularly relevant for performance-driven domains like eSports.

### General Self-Efficacy Scale—short version (GSE-3)

The General Self-Efficacy Scale—Short Version (GSE-3) measures individuals' confidence in their ability to manage tasks and challenges effectively. This study employed the Arabic version developed by (Fekih-Romdhane et al. 2023), which has shown excellent psychometric properties, including Cronbach's alpha of 0.93 and strong criterion validity. The scale includes three items; each rated on a 5-point Likert scale ranging from 1 (“do not agree at all”) to 5 (“completely agree”). The total score is calculated as the average of the three responses, yielding a score between 3 and 15, with higher values indicating greater perceived self-efficacy.

### Statistical analysis

All statistical analyses were conducted using SPSS software (version 27.0). Descriptive data are presented as means with Standard Deviations (SD) for continuous variables and percentages for categorical variables. Associations between categorical variables (e.g., gender, device type, prize winnings, and game type) were examined using Pearson's chi-square test (χ^2^), with Phi coefficient (ϕ) reported as the measure of effect size. The strength of association based on ϕ was interpreted as follows: >0 (negligible), >0.05 (weak), >0.10 (moderate), >0.15 (strong), and >0.25 (very strong). When significant associations were detected, Odds Ratios (OR) with 95% Confidence Intervals (CI) were calculated. To control for potential Type I error from multiple comparisons, a Bonferroni correction was applied to the bivariate analysis of gaming behavior variables (device type, tool used, frequency, and duration). As each body region in the NMQ-E is treated independently, the correction was applied across the four predictors, yielding an adjusted significance threshold of α = 0.0125. Associations below this threshold were considered statistically significant.

To explore relationships between categorical and continuous variables, Eta coefficients were used, with values interpreted as follows: 0–0.1 (none to negligible), 1–0.3 (weak), 3–0.5 (moderate), and >0.5 (strong), in line with Cohen's guidelines (2013). For continuous variables, Spearman's rank correlation was used to assess the strength and direction of associations. MANCOVA was used to assess the main effect of Musculoskeletal pain across five body regions on flow and self-efficacy outcomes, controlling for age. A *p-*value < 0.05 was considered statistically significant for all tests.

### Ethical considerations

Prior to participation, all respondents provided informed consent at the start of the questionnaire, confirming that their data would be collected solely for academic research purposes and handled with full confidentiality. The agreement was confirmed by clicking “Agree and complete the questionnaire”, which served as digital consent. The study received ethical approval from the Institutional Review Board at Princess Nourah Bint Abdulrahman University (IRB approval number: 24-0490).

## Results

A complete list of 370 professional gamers was obtained directly from SEF. After excluding twenty one individuals under the age of 18, a total of 349 eligible participants remained. All eligible individuals were approached using a census sampling approach, resulting in 232 completed responses and a response rate of 66.5%.

### Participant demographics

The study sample consisted of two hundred and thirty two male individuals, with an average age of 22.7 ± 4.5 years. A large proportion of participants reported residing in the Central Region (50.9%). Most respondents held a bachelor's degree (70.7%). The participants represented a range of employment situations, including students (53.9%), employed individuals (24.1%), and those who were unemployed (22%). Regarding marital status, the sample was primarily composed of individuals who were single (94.4%). Details are expressed in Table 1.

**Table 1:** Participants' personal and gaming characteristics.

**1-Region**	**Frequency (*n*) %**
Central region	118 (50.9%)
Eastern region	31 (13.4%)
Northern region	16 (6.9%)
Southern	6 (2.6 %)
Western region	61 (26.3%)
**2-Educational level**	**Frequency (** ***n*** **) %**
High School	63 (27.2 %)
Bachelor's degree	164 (70.7%)
Postgraduate Degree	5 (2.2 %)
**3-Employment status**	**Frequency** ***(n*****) %**
Employed	56 (24.1%)
Un-Employed	51 (22 %)
Student	125 (53.9 %)
**4-Game characteristics**	**Frequency (** ***n*** **) %**
PUBG Mobile	46 (19.8 %)
Mobile legend: bang bang	4 (1.7 %)
Multiplayer online battle arena	50 (21.6 %)
Overwatch 2	25 (10.8 %)
Rainbow six siege	27 (11.6 %)
Valorant	21 (9.1 %)
COD	50 (21.6 %)
First person shooter	123 (53%)
Rocket league	18 (7.8%)
EAFC 24	22 (9.5 %)
eFootball	18 (7.8 %)
Others	58 (25 %)
**5-Tournamenant participation**	**Frequency (** ***n*** **) %**
Yes	219 (94.4 %)
No	13 (5.6 %)
**6-Prize win status**	**Frequency (** ***n*** **) %**
Yes	153 (65.1 %)
No	79 (34.1 %)
**7-Preferred device**	**Frequency (** ***n*** **) %**
Desktop	106 (45.7 %)
Laptop	10 (4.3 %)
PlayStation	49 (21.1 %)
Smart device (phone-Tablet)	67 (28.9 %)
**8-Tool used**	**Frequency (** ***n*** **) %**
Keyboard and mouse	73 (31.9 %)
Controller	115 (49.6 %)
Touch	43 (18.5 %)
< 5 h	83 (35.3 %)
≥5 h	150 (64.7 %)
**10- Days played per week**	**Frequency (** ***n*** **) %**
1-4	35 (15.1%)
5-7	197 (84.9 %)

Study participants were registered with the SEF across nine different game genres, as detailed in Table 1. The majority (53.0%) competed in First-Person Shooter (FPS) games, followed by 21.6% in the Multiplayer Online Battle Arena (MOBA) category. A substantial proportion (94.4%) reported participating in eSports tournaments, and 65.1% of them had won prizes. Participants reported a high level of gaming engagement, with 84.9% playing 5 to 7 days per week, and 64.7% spending five or more hours daily on gameplay Table 1.

### Prevalence and consequences of Musculoskeletal pain

MSK pain was reported by 72% of participants. The neck was the most frequently affected region, cited by 38.4% of those reporting pain, followed by the lower back (31.5%), wrists and hands (24.1%), and upper back (22.8%). Pain in the neck and back regions demonstrated notable chronicity, with participants reporting an average pain duration of 4.3 ± 4.4 years for the neck and 3.5 ± 3.2 years for the lower back.

In terms of functional consequences, pain led to activity limitations across multiple regions. The most reported activity limitations were reported in the ankles/feet (57.9%) and knees (39.5%). Additionally, a substantial proportion of participants experienced activity limitations related to the upper back (39.6%), wrists and hands (37.7%), lower back (34.2%), and neck (33.7%), highlighting the widespread functional burden associated with MSK pain. These findings are summarized in Table 2.

**Table 2:** Musculoskeletal pain prevalence and consequences by body region and timeframe as assessed by the NMQ-E.

	**Timeframe pain prevalence**				**Impact of pain**		
**Pain site**	**Lifetime** ***N*** **(%)**	**Annal** ***N*** **(%)**	**Month** ***N*** **(%)**	**Poit** ***N*** **(%)**	**Hospitaliztion** ***N*** **(%)**	**Activity limitation** ***N*** **(%)**	**Average time from involvment (years) Mean** ***(SD*****)**
Neck	89 (38.4)	61 (26)	38 (16.4)	11 (4.7)	3 (3.4)	30 (33.7)	4.3 (4.5)
Shoulder	42 (18.1)	21 (9.1)	14 (6)	11 (4.7)	3 (7.1)	12 (28.6)	2.9 (2.4)
Upper back	53 (22.8)	32 (13)	24 (10.3)	14 (6)	1 (1.9)	21 (39.6)	3.1 (2.3)
Elbows	10 (4.3)	4 (1.7)	3 (1.3)	2 (0.9)	1 (10)	2 (20)	2.2 (1.8)
Wrist/hands	56 (24.1)	32 (13.8)	16 (6.9)	8 (3.4)	6 (10.7)	20 (37.7)	3.1 (2.5)
Low back	73 (31.5)	41 (17.7)	29 (12.5)	15 (6.5)	3 (4.1)	25 (34.2)	3.5 (3.2)
Hips/thighs	5 (2.2)	2 (0.9)	1 (0.4)	0 (0)	1 (20)	1 (20)	3 (3)
Knees	38 (16.4)	22 (9.5)	15 (6.5)	7 (3)	7 (18.4)	15 (39.5)	3.3 (3.1)
Ankle/feet	19 (8.2)	15 (6.5)	12 (5.2)	4 (1.7)	4 (21.1)	11 (57.9)	2.8 (3.6)

### Associations between personal and gaming factors with Musculoskeletal pain

Bivariate analyses revealed significant associations between specific gaming factors and the annual prevalence of musculoskeletal pain in certain body regions. Device type was significantly associated with wrist and hand pain (χ^2^ = 19.242, *df* = 6, *p* = 0.004, ϕ = 0.288), and tool used during gameplay (e.g., mouse, controller) also showed a significant relationship with wrist and hand pain (χ^2^ = 12.076, *df* = 4, *p* = 0.017, ϕ = 0.228). For shoulder pain, a significant association was observed with tool type (χ^2^ = 19.973, *df* = 4, *p* < 0.001, ϕ = 0.293). Additionally, the number of days played per week was significantly associated with both shoulder pain (χ^2^ = 6.648, *df* = 2, *p* = 0.036, ϕ = 0.169) and upper back pain (χ^2^ = 6.598, *df* = 2, *p* = 0.037, ϕ = 0.169). In contrast, no significant associations were observed between the number of hours played per day and pain prevalence in any region. After applying a Bonferroni correction to account for multiple comparisons across the four gaming behavior variables, only the associations between shoulder pain and tool used (*p* < 0.001) and wrist/hand pain and device type (*p* =0.004) remained statistically significant (α = 0.0125). These results are presented in Table 3.

**Table 3:** Association of annual pain prevalence by body location and sample characteristics.

**Variables**	***N***	**Device type**			**Tool used**			**Days played per week**			**Hours played per day**		
**TEST**		**X** ^2^	**Df**	***P***	**X** ^2^	***Df***	***P***	**X** ^2^	***Df***	***P***	**X** ^2^	***Df***	***P***
Neck pain	*N* = 61	3.942	6	0.685	2.405	*4*	0.662	4.984	2	0.083	0.785	2	0.675
Shoulder pain	*N* = 21	7.895	6	0.246	19.973	4	< 0.001^**^	6.648	2	0.036	0.107	2	0.948
Elbow	*N* =4	6.287	6	0.392	1.648	4	0.800	0.731	2	0.694	4.767	2	0.092
Wrist/Hand pain	*N* = 32	19.242	6	0.004^**^	12.076	4	0.017^*^	2.670	2	0.263	0.071	2	0.965
Upper back pain	*N* = 32	8.021	6	0.237	2.290	4	0.683	6.598	2	0.037	2.896	2	0.235
Lower back pain	*N* = 41	5.497	6	0.482	0.987	4	0.912	1.115	2	0.573	0.479	2	0.787
Hip	*N* = 2	1.298	6	0.972	2.830	4	0.587	0.908	2	0.635	1.110	2	0.574
Knee	*N* = 22	9.136	6	0.116	6.186	4	0.186	2.292	2	0.318	0.560	2	0.756
Ankle and Foot	*N* = 15	5.246	6	0.513	1.915	4	0.751	3.100	2	0.212	0.881	2	0.644

*N* = number of participants, X^2^ = Pearson's chi-squared; df = degrees of freedom. P^*^ = Level of significance, α =.05. ^*^p < 0.05, ^**^p < 0.0125 (Bonferroni corrected).

To further explore these associations, binary logistic regression analyses were conducted separately for each of the five primary musculoskeletal pain locations—neck, shoulder, upper back, lower back, and wrist/hand—using age, device type, tool used, and number of gaming days per week as predictors. Models for neck, lower back, and wrist/hand pain were not statistically significant (*p* > 0.05), and no individual predictors emerged as significant within those models. However, trend-level associations were observed in the neck pain model for age (*p* = 0.098) and gaming frequency (*p* = 0.071), suggesting potential patterns that may warrant further investigation. The model for shoulder pain was statistically significant (χ^2^(7) = 24.56, *p* < 0.001), explaining 59% of the variance (Nagelkerke *R*^2^ = 0.590). Given the strength and significance of this model, individual regression coefficients are reported: both input tool (Wald = 4.72, *p* =.030) and device category (Wald = 5.56, *p* = 0.018) were significant predictors. Although the association was statistically significant (*OR* = 12.83), the wide 95% confidence interval (1.27–130.18) suggests substantial uncertainty, likely due to a small number of controller users, which may limit the stability of the estimate. For upper back pain, the model approached significance (χ^2^(7) = 13.02, *p* =.072), with one device category emerging as a significant predictor (p =.048). Across all models, Hosmer–Lemeshow tests indicated good model fit. Collectively, these findings suggest that while certain gaming characteristics may influence MSK pain in specific anatomical regions—particularly the shoulder and upper back—these factors do not universally predict pain presence across all regions.

Table 4 presents the logistic regression results for shoulder pain. In this model input tool and device category were significant predictors. Using a keyboard instead of a controller (reference category) was associated with significantly higher odds of reporting shoulder pain [*B* = 2.552, *p* = 0.030, *OR* = 12.83, 95% *CI* (1.27, 130.18)]. Other input methods, including touchscreens and combined tools, did not significantly differ from controller users. In terms of device type, gamers using a smart device had significantly lower odds of experiencing shoulder pain compared to those using a desktop (reference) [*B* = −1.438, *p* = 0.018, *OR* = 0.237, 95% *CI* (0.07, 0.80)], suggesting a potential protective effect. Use of laptops and PlayStation consoles did not significantly differ from desktop use in predicting shoulder pain. Age (*p* = 0.226) and the number of gaming days per week (*p* = 0.279) were also not significant predictors. Overall, the model was statistically significant (χ^2^(7) = 24.56, *p* < 0.001), explained a substantial proportion of variance (Nagelkerke *R*^2^ = 0.590), and demonstrated good fit based on the Hosmer–Lemeshow test. These results indicate that both the type of input tool and device used during gaming may influence the risk of shoulder pain, with keyboard use appearing riskier and smart device use possibly less so when compared to standard desktop setups.

**Table 4:** Logistic regression results for shoulder pain.

**Predictor**	***B***	***SE***	**Wald**	***p*-value**	***OR* (Exp(B))**	**95% CI lower**	**95% CI upper**
Age	−0.037	0.03	1.525	0.226	0.964	0.9	1.032
Tool: controller vs. mouse	2.552	1.177	4.719	0.03	12.833	1.265	130.183
Tool: other vs. mouse	0.346	1.146	0.091	0.761	1.412	0.163	12.222
Device: category 1 vs. PC	1.418	1.043	1.849	0.173	4.13	0.518	32.926
Device: Category 2 vs. PC	−1.438	0.609	5.559	0.018	0.237	0.07	0.802
Days per week	0.547	0.501	1.192	0.279	1.728	0.642	4.651

### Flow state, self-efficacy levels, and their association with Musculoskeletal pain

Descriptive statistics for the Flow State Scale and the General Self-Efficacy Scale are presented in Table 5. Scores were interpreted based on conventional cutoffs for 5-point Likert scales, where values ≥3.5 are typically considered moderate to high (Boone and Boone, 2012). Participants reported moderate to high levels of flow and self-efficacy. Among the flow dimensions, Flow Cognitive and Flow Wellbeing had the highest mean scores (*M* = 3.7, *SD* = 1.2; Median = 4), suggesting strong cognitive engagement and a positive sense of well-being during gameplay. Flow Time and Flow Ego yielded slightly lower mean scores (*M* = 3.5, *SD* = 1.2–1.3; Median = 3.75), still falling within the moderate to high range. Similarly, the mean self-efficacy score was 3.6 (*SD* = 1.3, Median = 4), indicating generally confident self-perceptions among participants.

**Table 5:** Flow status, self-efficacy and the association with Musculoskeletal pain.

**Pain site**	**Flow cognitive**	**Flow time**	**Flow ego**	**Flow wellbeing**	**Self-efficacy**
Mean (SD)	3.7 (1.2)	3.5 (1.2)	3.5 (1.3)	3.7 (1.2)	3.6 (1.3)
Median	4	3.75	3.75	4	4
**Test**	**Eta**				
Neck pain	0.017	0.121	0.014	0.007	0.005
Shoulder pain	0.025	0.093	0.097	0.064	0.039
Elbow pain *N* =	0.010	0.041	0.025	0.029	0.027
Wrist/hand pain=	0.144	0.071	0.103	0.058	0.112
Upper back pain	0.016	0.061	0.034	0.007	0.010
Lower back pain	0.009	0.084	0.006	0.051	0.029
Hip pain	0.040	0.048	0.021	0.050	0.061
Knee pain	0.058	0.094	0.055	0.103	0.050
Foot and ankle	0.020	0.060	0.025	0.091	0.032

Eta (0 indicating no association, 1 indicating perfect association).

Eta value was used to assess the strength of the association between MSK Pain, Flow State, and Self-Efficacy. The results indicated no association with the presence of pain, self-efficacy, and flow states in all domains (Table 5).

To further explore whether pain in specific body regions affected psychological outcomes, MANCOVA was conducted. The main effect of musculoskeletal pain (pooled across five primary anatomical regions: neck, shoulder, wrist/hand, upper back, and lower back) on the combined dependent variables—Flow Cognitive, Flow Ego, Flow Time, Flow Well-being, and Self-Efficacy—was examined, while controlling for age. Box's M test was significant (*p* = 0.003), indicating unequal covariance matrices; therefore, Pillai's Trace was used due to its robustness to this violation. The multivariate test did not reach statistical significance; Pillai's Trace = 0.044, F (5, 225) = 2.078, *p* = 0.069, partial η^2^ = 0.044. Despite the non-significant *p*-value, the partial η^2^ suggests a potentially meaningful effect in practical terms, consistent with a small-to-medium impact. This suggests that the presence of site-specific musculoskeletal pain did not result in meaningful differences across the psychological outcomes when considered jointly. Univariate between-subjects analyses further confirmed the lack of significant differences across individual outcomes (Table 6). None of the flow subdomains or self-efficacy were significantly associated with musculoskeletal pain in the five primary sites. All *p*-values exceeded the.05 threshold, and partial η^2^ values were uniformly low, indicating negligible effect sizes across individual outcomes. However, it is worth noting that the overall MANCOVA model yielded a partial η^2^ of.044, suggesting a small-to-medium multivariate effect that may be of practical interest despite the non-significant p-value. These findings reinforce earlier bivariate results using Eta, demonstrating that localized musculoskeletal pain does not meaningfully impact psychological flow or self-efficacy among professional gamers.

**Table 6:** Univariate effects of pain on flow and self-efficacy (MANCOVA, Age-Controlled).

**Dependent variable**	***F***	***p***	***η^2^***
Flow cognitive	0.901	0.344	0.004
Flow time	1.654	0.200	0.007
Flow ego	0.701	0.403	0.003
Flow wellbeing	0.087	0.768	0.000
Self-efficacy	0.174	0.677	0.001

*MANCOVA* = Multivariate Analysis of Covariance; F = F-statistic; *p* = significance level; η^2^ = partial eta squared, a measure of effect size.

## Discussion

The present study investigated the prevalence and consequences of MSK pain among professional gamers in Saudi Arabia, exploring its potential association with flow states and self-efficacy during gameplay. A substantial proportion of participants reported MSK pain, particularly in the upper body and back areas, commonly strained during prolonged gaming. Despite this high burden of physical symptoms, gamers demonstrated strong psychological engagement across all four dimensions of flow—cognitive involvement, time distortion, ego detachment, and welbeing—as well as elevated levels of self-efficacy. Notably, no significant associations were found between MSK pain and these psychological variables, suggesting that flow and self-efficacy may remain unaffected even in the presence of discomfort. These findings highlight the potential psychological resilience of professional gamers; a population uniquely adapted to high cognitive and physical demands.

In total, 72% of participants reported musculoskeletal pain, with symptoms affecting all major body regions. The most commonly reported sites included the neck, upper back, upper extremities, and lower back, consistent with postural strain and repetitive-use syndromes described in occupational health literature. These patterns reflect those reported in prior studies of eSports athletes. For instance, research from Denmark revealed a high prevalence of musculoskeletal pain among competitive gamers, particularly in the neck, shoulders, back, and wrists (Lindberg et al., 2020; Sand Hansen et al., 2025). Similarly, (Chung et al. 2022) identified neck and finger pain as common complaints among elite mobile eSports players, largely attributed to prolonged gaming duration and inadequate ergonomic conditions.

In the present study, both the gaming platform and input tools (e.g., controllers, mice, key boards) were initially found to be significantly associated with shoulder and wrist/hand pain. However, after applying a Bonferroni correction to adjust for multiple comparisons, only input tool type remained significantly associated with shoulder pain, and device type with wrist/hand pain—underscoring the influence of hardware design and interaction style. These findings are supported by prior literature linking musculoskeletal discomfort to posture, intensity, and device type (Kar et al., 2024; Ahmadi Shoar et al., 2025). For example, (Tiric-Campara et al. 2014) noted that prolonged mouse and keyboard use may lead to shoulder stiffness due to static positioning. In contrast, (Phuah Rong Yao et al. 2021) found that mobile device users often experienced pain related more to posture than equipment constraints. Collectively, these results underscore the importance of ergonomic awareness and the need for tailored interventions based on platform type. The strong association between keyboard use and shoulder pain should be interpreted with caution due to the wide confidence interval, likely reflecting limited sample size in the controller group. While this result aligns with ergonomic concerns in eSports, future studies with more balanced representation across input-device types are needed to confirm this trend.

Psychologically, participants reported high scores in both flow and self-efficacy, with particularly elevated ratings in the wellbeing and cognitive dimensions of flow. These findings align with previous studies highlighting the performance-enhancing and emotionally rewarding aspects of flow in eSports contexts (Chalghaf et al., 2019; Martiny et al., 2023). Furthermore, self-efficacy was consistently high, supporting prior research suggesting that competitive gaming enhances perceived control, confidence, and persistence (Gutierrez, 2021; Chung et al., 2020).

Interestingly, no statistically significant associations were observed between the presence of pain and any individual flow or self-efficacy outcomes. However, the overall pattern of results suggests that physical discomfort may exert a subtle influence on psychological functioning. Although these effects did not reach the conventional threshold for statistical significance, they were reflected in small effect sizes and may remain undetected when outcomes are analyzed individually. This partially aligns with (Ditchburn et al. 2020), who found that immersive task engagement during exergaming could reduce perceived pain. While the present study did not demonstrate pain reduction, it revealed an important dissociation between physical discomfort and psychological engagement.

The ability of gamers to maintain high flow and self-efficacy scores despite reported MSK pain may point to underlying self-regulatory strategies that preserve performance under physical strain. These findings can be better understood through the lens of three theoretical frameworks introduced earlier: the biopsychosocial model, Attentional Control Theory, and Self-Efficacy Theory. From a biopsychosocial perspective (Gatchel et al., 2007), the psychological response to pain is shaped by cognitive and motivational factors that may mitigate its impact on performance-related functioning. Despite the potential for pain to disrupt attention and immersion (Eysenck et al., 2007), the consistently high flow scores in our sample suggest that professional gamers may have developed compensatory attentional strategies or cognitive adaptations that help maintain focus during gameplay.

Likewise, the high self-efficacy scores observed among participants may reflect well-developed task-related beliefs built through extensive competitive experience (Bandura, 1997). This suggests that even in the presence of physical symptoms, elite gamers retain strong performance confidence, possibly due to habitual coping, goal prioritization, or motivational regulation. These interpretations highlight the complex and potentially non-linear relationship between physical strain and psychological functioning in eSports and underscore the need for future studies to explore mediating factors such as pain severity, coping style, or intrinsic motivation.

These results reinforce the notion that eSports athletes may possess cognitive mechanisms, such as attentional absorption or motivational regulation, that help buffer psychological functioning against physical discomfort. This warrants further research into the neurocognitive and emotional regulation strategies that support sustained performance under physical stress. Moreover, the observed coexistence of pain and optimal mental states suggests that long-term physical consequences may go unnoticed or unaddressed by players or stakeholders. This raises concerns about the sustainability of high performance and the risk of burnout in the professional gaming industry.

This study has several limitations. Its cross-sectional design limits the ability to draw causal inferences. Self-reported data are subject to recall and social desirability biases, and the use of Likert-type response formats without reverse-coded items may have limited reduced sensitivity to response inconsistency or inattentiveness. Furthermore, completion time was not recorded or analyzed, which limited the ability to identify careless responses or control for systematic variation in response patterns. Additionally, the study sample consisted exclusively of male professional gamers due to the gender composition of the official registry at the time of data collection. As such, the findings may not be generalizable to female gamers or more gender-diverse eSports communities. Future research should aim to include more inclusive and representative samples as the eSports field continues to evolve.

Additionally, factors such as pain severity, duration, and lifetime gaming exposure were not assessed. Future research should employ longitudinal designs, objective biomechanical and physiological data, and diverse samples to explore the mechanisms and outcomes of this physical-psychological interaction more comprehensively.

## Conclusion

This study provides important insight into the high prevalence of musculoskeletal pain among professional gamers in Saudi Arabia, with the upper body being most affected. Despite these physical challenges, participants maintained high levels of flow and self-efficacy, suggesting that psychological functioning may be preserved through mechanisms such as attentional focus, motivational control, or emotional desensitization. These findings highlight the complex interplay between physical discomfort and mental performance in eSports, where psychological attributes can remain intact even under physiological strain. However, the absence of protective or buffering effect of pain highlights the need for integrated strategies—combining ergonomic, rehabilitative, and mental skills training—to support both performance and long-term health. Continued research is essential to guide evidence-based interventions and ensure the sustainability of elite gaming careers.

## Data Availability

The original contributions presented in the study are included in the article/supplementary material, further inquiries can be directed to the corresponding author.
